# Improved transfer efficiency of supercharged 36 + GFP protein mediate nucleic acid delivery

**DOI:** 10.1080/10717544.2022.2030430

**Published:** 2022-01-25

**Authors:** Lidan Wang, Jingping Geng, Linlin Chen, Xiangli Guo, Tao Wang, Yanfen Fang, Bonn Belingon, Jiao Wu, Manman Li, Ying Zhan, Wendou Shang, Yingying Wan, Xuemei Feng, Xianghui Li, Hu Wang

**Affiliations:** a Department of Microbiology and Immunology, Medical School, China Three Gorges University, Yichang, China; b Hubei Key Laboratory of Tumor Microenvironment and Immunotherapy, China Three Gorges University, Yichang, China; c Affiliated Ren He Hospital of China Three Gorges University, Yichang, China; d The First Clinical Medical College of China Three Gorges University, Yichang, China; e College of Biological and Pharmaceutical Sciences, China Three Gorges University, Hubei, China; f School of Medicine, Institute of Cell Engineering, Johns Hopkins University, Baltimore, MD, USA

**Keywords:** Cell-permeable peptides (CPPs), supercharged protein, 36 + GFP, Dot1l, plasmid DNA delivery

## Abstract

The potential of nucleic acid therapeutics to treat diseases by targeting specific cells has resulted in its increasing number of uses in clinical settings. However, the major challenge is to deliver bio-macromolecules into target cells and/or subcellular locations of interest ahead in the development of delivery systems. Although, supercharged residues replaced protein 36 + GFP can facilitate itself and cargoes delivery, its efficiency is still limited. Therefore, we combined our recent progress to further improve 36 + GFP based delivery efficiency. We found that the penetration efficacy of 36 + GFP protein was significantly improved by fusion with CPP-Dot1l or treatment with penetration enhancer dimethyl sulfoxide (DMSO) *in vitro*. After safely packaged with plasmid DNA, we found that the efficacy of *in vitro* and *in vivo* transfection mediated by 36 + GFP-Dot1l fusion protein is also significantly improved than 36 + GFP itself. Our findings illustrated that fusion with CPP-Dot1l or incubation with DMSO is an alternative way to synergically promote 36 + GFP mediated plasmid DNA delivery *in vitro* and *in vivo*.

## 1. Introduction

Currently, most of medicines on the pharmacies are small chemical molecules, which were manufactured by chemical synthesis. Small molecules are used to treat a wide variety of human diseases and conditions as demonstrated by the increasing approved nucleic therapeutics by the Food and Drug Administration (FDA). For example, insulin used in treating diabetic patients, and the world’s first mRNA vaccine – the COVID-19 vaccines from Pfizer/BioNTech and Moderna are remarkably potent against SARS-Cov-2 infection both utilizing components of nucleic acid therapeutics (Kim et al., 2021; Oliver et al., 2021; Pushparajah et al., 2021). The size, complexity, and stability of biological therapeutic macromolecules can result in highly target specificities compared to small molecule drugs (Zhang et al., 2018). However, the large size of the macromolecules makes them difficult to diffuse into cells, thus resulting in all existing therapeutic biologics target extracellularly and not intracellularly. Therefore, how to deliver bio-macromolecules into target cells and/or subcellular locations of interest is a major challenge ahead in the development of delivery systems.

To address this challenge, a variety of delivery approaches have been developed by multidisciplinary research communities, including physical (thermoporation, electroporation, magnetoporation, mechanoporation, optoporation, sonoporation, and microinjection) strategies (Meacham et al., 2014; Du et al., 2018), chemical strategies (artificial lipids (Hou et al., 2022), dendrimers (Lu et al., 2019), polymers (Farshbaf et al., 2018), carbon nanotubes (Mangla et al., 2020) or nanoparticles and direct chemical modification such as PLGA (Matta & Maalouf, 2019) or cholesterol (Ruwizhi & Aderibigbe, 2020)), and biological strategies (viral delivery (Lundstrom, 2020) and non-viral delivery (Bono et al., 2020) such as cell penetrating peptides (CPPs)) (Thompson et al., 2012; Liu et al., 2016; Geng et al., 2021).

Cell penetrating peptides are short cationic peptides (examples include Tat (Wang et al., 2010; Wu et al., 2018) and Dot1l (Geng et al., 2020)), that facilitate cellular uptake of molecular cargoes (Kardani et al., 2019), including small chemical compounds (Uhl et al., 2020; Khan et al., 2021), peptides, proteins (Suresh et al., 2017), DNA/RNA (Shukla et al., 2014; Kato et al., 2016; Geng et al., 2021), nanoparticles (Berry, 2008), and liposomes (Liu et al., 2016). CPPs also have been proven to have the ability to deliver cargoes to pass biological barriers (like skin (Schutze-Redelmeier et al., 2004) and/or mucosal (Ji et al., 2017)), it is worth mentioning that several promising vaccine delivery systems based on CPPs have been developed. However, references suggested that the use of CPPs was often not sufficient for vaccine efficacy (Skwarczynski & Toth, 2019; Yang et al., 2019). Thus, this approach is still underdeveloped and require additional study.

In recent years, supercharged protein, a class of naturally or engineered proteins with unusually high net positive negative charge, has attracted great interest because of its permeability in *in vitro* and *in vivo* (Thompson et al., 2012; Mangla et al., 2020). All engineered supercharged green fluorescent proteins (GFPs, net theoretical charges: −30 to +48) exhibit nearly identical emission and excitation spectrum as starting GFP. References suggested that 36 + GFP can enter the cells and mediate fusion protein (Motevalli et al., 2018) and siRNA (McNaughton et al., 2009) delivery into cultured cells *in vitro* and the inner ear of live mice *in vivo* (Li et al., 2015; Zhang et al., 2020); however, we noted that the efficiency of 36 + GFP based delivery was very low and not well-distributed in cytosol (Wu et al., 2015). Moreover, the majority of 36 + GFP protein was entrapped in the endosome (Thompson et al., 2012); although, the Aurein 1.2 as an antimicrobial peptide (AMP) can facilitate the endosomal escape of a variety of proteins fused to 36 + GFP *in vitro*, it only achieves 50% efficiency (Li et al., 2015). Hence, our aim is to identify a way to increase the efficiency of 36 + GFP based delivery. Recently, we found that some of the penetration enhancers can be used to enhance the penetration efficiency of CPPs (Liu et al., 2016; Geng et al., 2021), and we also identified some novel CPPs like Dot1l (Geng et al., 2020), which can efficiently enter the cultured cells itself and can mediate fusion cargoes like GFP delivery without the help of penetration enhancers. Thus, we hypothesized that the delivery efficacy of supercharged 36 + GFP fused with Dot1l can be further improved.

In this study, we designed 36 + GFP-Dot1l fusion protein, generated in bacterial expression system, and its penetration efficacy was evaluated *in vitro* and *in vivo*. We also examined its plasmid DNA packaging, and further investigated its ability of mediating plasmid transfection *in vitro* and *in vivo*. We found that the efficacy of *in vitro* and *in vivo* transfection mediated by 36 + GFP-Dot1l fusion protein is significantly improved than 36 + GFP itself. These findings suggested that CPPs can synergically promote 36 + GFP mediated plasmid delivery *in vitro* and *in vivo*, which may lay a foundation for supercharged 36 + GFP protein based macromolecular delivery in future.

## 2. Materials and methods

### 2.1. Protein purification and cell culture

The well-constructed recombinant plasmids pET15b-GFP, pET15b-GFP-Dot1l, pET15b-36 + GFP, and pET15b-36 + GFP-Dot1l (36+GFP fragment were amplified through plasmid of Aurein1.2_(+36GFP)-Cre ordered from Addgene (number #71748)) were transformed in *E. coli* strain BL21 (DE3). GFP, GFP-Dot1l, 36 + GFP, and 36 + GFP-Dot1l fusion protein expressions (DNA and protein sequence are attached in Table S1) were induced with 0.1 mM isopropyl-β-d-thiogalactoside (IPTG) at 37 °C for 4 h. After bacteria were harvested by centrifugation, it was resuspended in lysis buffer containing 10 mM Tris pH 8.0, and then lysed by ultrasonication. The fusion proteins were purified by Ni-NTA affinity chromatography (Qiagen, Hilden, Germany) with a purification buffer consisting of washing solution (1 M NaCl, 10 mM Tris, 20 mM imidazole), elution solution (1 M NaCl, 10 mM Tris, 200 mM imidazole), and equilibration solution (1 M NaCl, 10 mM Tris). After gradient dialysis, ultrafiltration and concentration measurement were conducted, and purified proteins were transferred to Eppendorf tubes store at −80 °C until further use.

Human breast cancer cell lines MCF7, human hepatocellular carcinoma-derived HepG2, and rat hepatic stellate cell line HSC-T6 cells were routinely maintained in our lab. All cell lines were grown in Dulbecco’s modified Eagle’s medium (DMEM) plus with 10% heated-inactivated fetal bovine serum (FBS) and 1% penicillin–streptomycin at 37 °C and 5% CO_2_.

### 2.2. Cellular uptake analysis

MCF7, HepG2, and HSC-T6 cells in logarithmic growth phase were collected and seeded in 24-well plates at a density of 1.6 × 10^5^ cells/well for culture. After the cells were cultured for 24 h, cells were washed twice with PBS, and four purified fusion proteins were added at a concentration of 5 µM with or without dimethyl sulfoxide (DMSO) pretreatment for 0.5 h. After incubation was over, the cells were washed three times with phosphate-buffered saline (PBS) and then imaged by fluorescence microscopy (Nikon, Tokyo, Japan).

To quantify the cell-penetration efficiency, a multi-mode spectrophotometry was used. Incubation and washing steps were performed as above. Cells were then lysed with 300 μl/well of lysis buffer radioimmunoprecipitation assay (RIPA) buffer with phenylmethylsulfonyl fluoride (PMSF) on ice for 20 min and centrifuged at 1000 rpm for 5 min. The fluorescence intensity of supernatant was read by using multi-mode spectrophotometry (Tecan, Mannedorf, Switzerland) at 485 nm excitation and 535 nm emission. The protein concentration of the supernatant was measured with the bicinchoninic acid (BCA) protein assay kit according to the manufacturer's recommendation. The fluorescence of cellular uptake was expressed as fluorescence intensity per mg of total cellular protein. The experiments mentioned in the text were repeated at least three times.

### 2.3. Western blotting

After treatment shown above was over, cells were washed with PBS three times and were lysed with RIPA lysis buffer supplemented with the protease inhibitor (PMSF) and incubated on ice for 30 minutes. Supernatant was collected after centrifugation (12,000 rpm, 15 min), and protein concentration was determined by using the BCA protein assay kit according to the manufacturer's recommendations. The lysate was boiled with loading buffer and separated with 10% sodium dodecyl sulfate polyacrylamide gels (SDS-PAGE) and transferred to polyvinylidene fluoride (PVDF) membranes. After blocking with blocking buffer (5% skim milk powder in TBST (0.1% Tween 20 in Tris-buffered saline (TBST)) for 1 h, primary antibody 6 × HisTag (mouse polyclonal, Cell Signaling Technology, Boston, MA; 1:1000) was incubated overnight at 4 °C. After washing PVDF membrane three times with TBST, goat anti-mouse secondary antibody (Santa Cruz Biotechnology, Dallas, TX; 1:1000) coupled with horseradish peroxidase (HRP) was incubated for another 1 h at room temperature. Anti-β-actin-HRP (Santa Cruz Biotechnology, Dallas, TX; 1:1000) was used as a loading control. Chemical reaction light signal detection was performed using the Clinx ChemiScope 3000 mini enhanced chemiluminescence (ECL) detection reagent.

### 2.4. *In vivo* penetration and transfection experiments of four proteins

All animal experiments were compliant with the National Research Council Guide for the Care and Use of Laboratory Animals, and the animal experimental protocol was approved by the Institutional Animal Care and Use Committee of China Three Gorges University. All our efforts were made to minimize the number of mice used and their suffering. In this study, 4–6 weeks old CD1 mice were obtained from our experimental animal center affiliated in China Three Gorges University. Animals were maintained at temperature (23 ± 2 °C) controlled and light-controlled (12-hour light per dark cycle) environment with free-access to food and water *ad libitum*.

We gently grab the tail of CD1 mice and pull it into the mouse restrainer, with leaving its tail sticking out of the small opening in the back of the restrainer. The four fusion proteins GFP, GFP-Dot1l, 36 + GFP, and 36 + GFP-Dot1l or relative protein/complex were injected into the mice by tail vein injection. After injection, the mice were released from the restrainer and were returned to the cage for another five hours (penetration assay) or 48 hours (transfection assay). Mice were perfused intracardially under deep pentobarbital anesthesia with ice-cold PBS followed by 4% paraformaldehyde (PFA) in PBS. Each organ was extracted and post-fixed overnight. After washing with PBS and dehydrated with 30% sucrose for 24 h, tissue or organ was embedded in cryomolds with OCT (Tissue-Tek, Torrance, CA) before freezing. Cryosections with 8–10 µm thick were cut from by using cryostat (Dakewei, Shenzhen, China) and mounted on the slide. Nuclei were counterstained with DAPI (1:5000) before imaging under a fluorescent microscope (SOPTOP).

### 2.5. Gel retardation assay

GFP, GFP-Dot1l, 36 + GFP, and 36 + GFP-Dot1l fusion proteins and DsRed-expressing plasmids were mixed in different mole ratios (0:1, 10:1, 50:1, 100:1, 200:1, 400:1, 800:1, and 1600:1) with 5 × DNA-protein binding buffer (50 mM Tris, 5 mM EDTA, 0.5 M KCl, 0.5 mM DTT, 25% v/v glycerol, and 0.05 mg/ml BSA) (Sidorova et al., 2010) with ddH_2_O to a final volume of 15 μl at room temperature for 20 min. Samples were supplement with loading buffer (0.1 M Tris–HCl PH 6.8, 50% glycerol, 0.04% bromophenol blue), and pre-electrophoresis on 5% native-PAGE gels with 100 V for 1 h, and further 60 V electrophoresis for 4 h. Then, the gels were stained with ethidium bromide (EtBr) for 15 min and imaged using the Kodak Gel Logic 2200 Imaging System (Rochester, NY).

To examine the complex stability in serum containing environment after protein plasmid complex was formed, 50% of FBS was added for another 2 or 4 hours at room temperature. Then, the sample preparation, PAGE running and imaging for serum stability of recombinant protein plasmid complex are nearly the same with protocol shown above. All samples mobility were presented by the distance of band away from the loading wells divided by the distance of control group. The experiments were repeated at least three times.

### 2.6. Zeta-potential and particle size measurement

The zeta-potential and particle size of protein plasmid complexes in different mole ratios (0:1, 100:1, 200:1, 400:1, 800:1, and 1600:1) were conducted by using a Zetasizer (Zetasize-Nano ZS90; Malvern Instruments, Worcestershire, UK), and all data were analyzed using Zetasizer software 6.30.

### 2.7. Hemolysis assay

The red blood cells (RBCs) were collected from CD1 mouse by centrifugation (1000 rpm, 5 min), after washing three times with PBS, and the RBCs were resuspended with PBS to prepare a 20% (v/v) RBCs suspension, 25 µl of RBCs suspension were incubated with protein/plasmid complex with different mole ratios (0:1, 100:1, 400:1, and 800:1) for 2 h in warm bath at 37 °C. The supernatant was removed after centrifugation for 5 min at 500 rpm, and 50 µl of supernatant was transferred to 96-well plate to read hemoglobin absorbance at 450 nm. The negative and positive control (incubated with 0.1% Triton X-100) were contained. The experiments were repeated at least three times.

### 2.8. Cytotoxicity assay

Cytotoxicity was measured using 3-(4,5-dimethylthiazol-2-yl)-2,5-diphenyl-2H-tetrazolium bromide (MTT) assay. MCF7 cells were seeded at a density of 8000 cells/well in 96-well culture plates. After 24 h culture, the supernatant was discarded, and the cells were washed with PBS and incubated with different mole ratio (0:1, 100:1, 400:1, and 800:1) of recombinant protein/pDNA complexes for 1 h. Then, the cells were washed with PBS and supplied with normal culture medium and incubated in incubator for 24 h or 48 h. After washing with PBS, 20 µl of 5 mg/ml MTT plus 80 µl serum containing medium were added into wells and incubated for another 4 h. Finally, the supernatant was discarded and 150 µl of DMSO was added to each well to dissolve the formazan crystals. The absorbance was read at 490 nm using a Multiskan Spectrum reader (Thermo Fisher Scientific, Waltham, MA). The experiments were repeated at least three times.

### 2.9. Lactate dehydrogenase leakage assay

MCF7 cells were seeded at a density of 8000 cells/well in a 96-well culture plate. After 24 h culture, each well was washed with 100 μl PBS and the wells were incubated with different mole rates (0:1, 100:1, 400:1, and 800:1) of recombinant protein/pDNA complexes for 2 h, then the supernatant was collected and incubated with reagent for LDH release assay (Beyonce) for 30 min. Following the protocol of manufacturer's recommendation, the absorbance of supernatant was read by a Multiskan Spectrum reader (Thermo Fisher Scientific, Waltham, MA) at the wavelength of 590 nm. The experiments were repeated at least three times.

### 2.10. Transfection evaluation

HSC-T6 and MCF7 cells were seeded into 24-well plates at a density of 1.6 × 10^5^ cells/well and cultured for 24 hours. The supernatant was discarded, and the cells were washed three times with PBS, and pretreated with 5% DMSO for 30 min. Different mole rates (0:1, 10:1, 50:1, 100:1, 200:1, 400:1, 800:1, 1600:1, and 2400:1) of recombinant protein/pDNA complexes were gently added into wells. After 4 h of incubation, 300 μl of normal culture medium was added into plates and incubated for 24 or 48 h. After washing with PBS, the recombinant protein-based transfection was observed under a fluorescent microscope (Nikon, Tokyo, Japan). TurboFectin (OriGene, Beijing, China) was used as a positive transfection reagent.

### 2.11. Statistical analysis

All present values from control and experimental group are expressed as means ± standard deviation (SD). Significance analysis (*p*< .05 was considered significant) was performed using GraphPad software Prism 7.0 (GraphPad Software, San Diego, CA).

## 3. Results

### 3.1. Recombinant protein purification and identification

**Figure 1.**
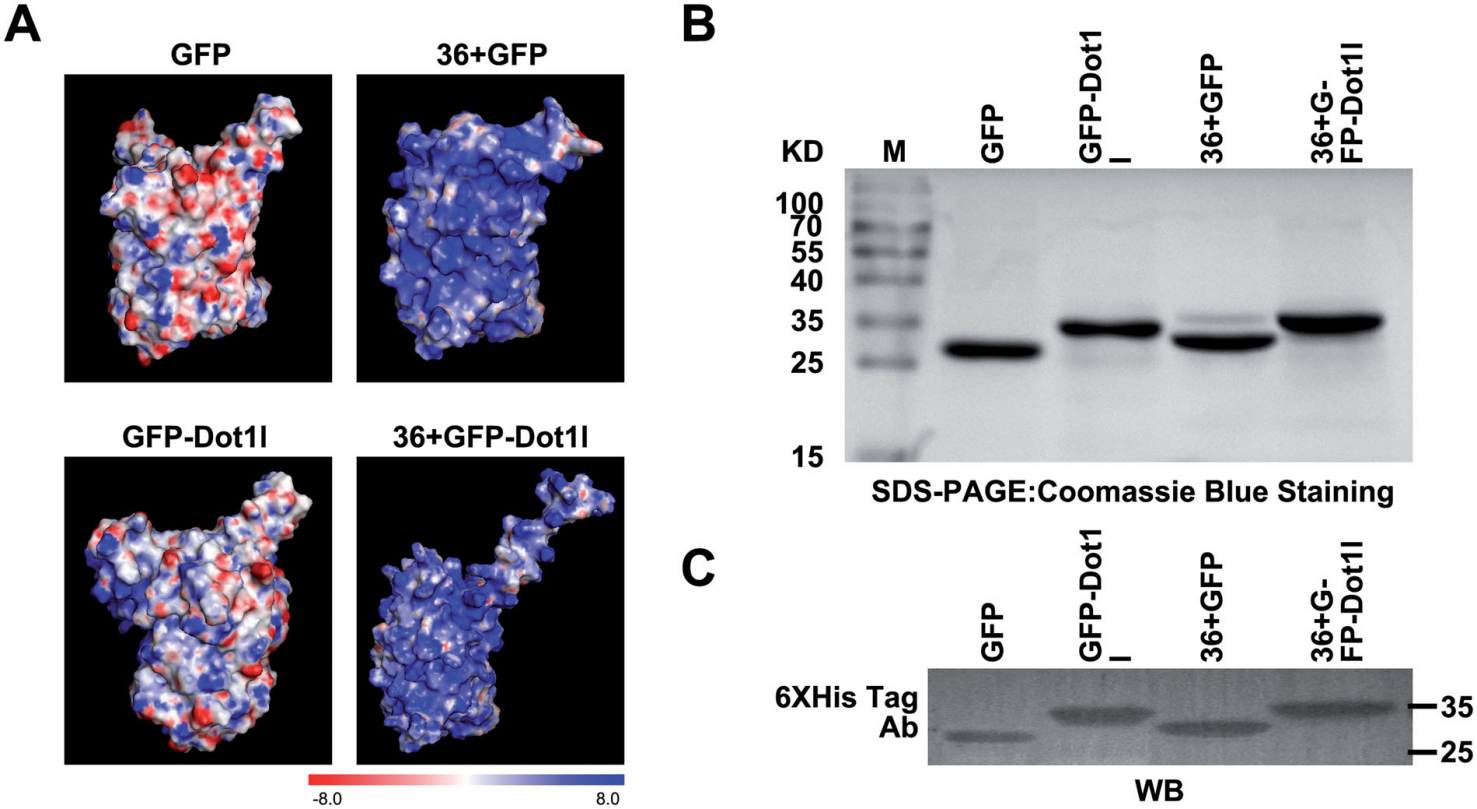
Overview of surface potential, expression and identification of recombinant protein used in this study. (A) Electrostatic surface potential of GFP, GFP-Dot1l, 36 + GFP, and 36 + GFP-Dot1l used in this study as calculated using the linearized Poisson–Boltzmann equation with APBS (Adaptive Poisson–Boltzmann Solver) from –8kT/e to +8kT/e. Red – negative charge, blue – positive charge. (B) Coomassie Blue stained SDS-PAGE analytical image showing purified GFP, GFP-Dot1l, 36 + GFP, and 36 + GFP-Dot1l recombinant protein expression in *E. coli*. (C) Western blotting analysis of purified GFP, GFP-Dot1l, 36 + GFP, and 36 + GFP-Dot1l recombinant protein which was probed with mouse monoclonal anti-6xhis antibody.

The overall positive charge plays a fundamental role in governing permeability of the proteins; however, translocation efficiency of supercharged 36 + GFP was still limited (Li et al., 2015), and its intracellular distribution did not distribute well. The structures and the calculated electrostatic surface potentials of GFP, GFP-Dot1l, 36 + GFP, and 36 + GFP-Dot1l are shown in Figure 1(A). We only can observe slightly different distribution of surface positive charge between GFP and GFP-Dot1l; however, there are subtle differences in the charge density and distribution in 36 + GFP and 36 + GFP-Dot1l. To address whether positive charge riches in Dot1 can affect the brightness of 36 + GFP, we used prokaryotic expression system to prepare recombinant His-tagged fusion protein GFP, GFP-Dot1l, 36 + GFP, and 36 + GFP-Dot1l, CPP-Dot1l fusion with GFP or 36 + GFP did not alter the brightness level of fluorescence (Figure S1A). Coomassie Blue stained SDS-PAGE analytical image showed that the expressed recombinant GFP, GFP-Dot1l, 36 + GFP, and 36 + GFP-Dot1l protein bands were located at the theoretically expected molecular weight (Figure 1(B)). Furthermore, to confirm the identity of purified protein bands, all these four fusion proteins were assessed by immunoblotting using the His-tag antibody (Figure 1(C)).

### 3.2. Recombinant protein efficiently permeates into cultured cells

**Figure 2.**
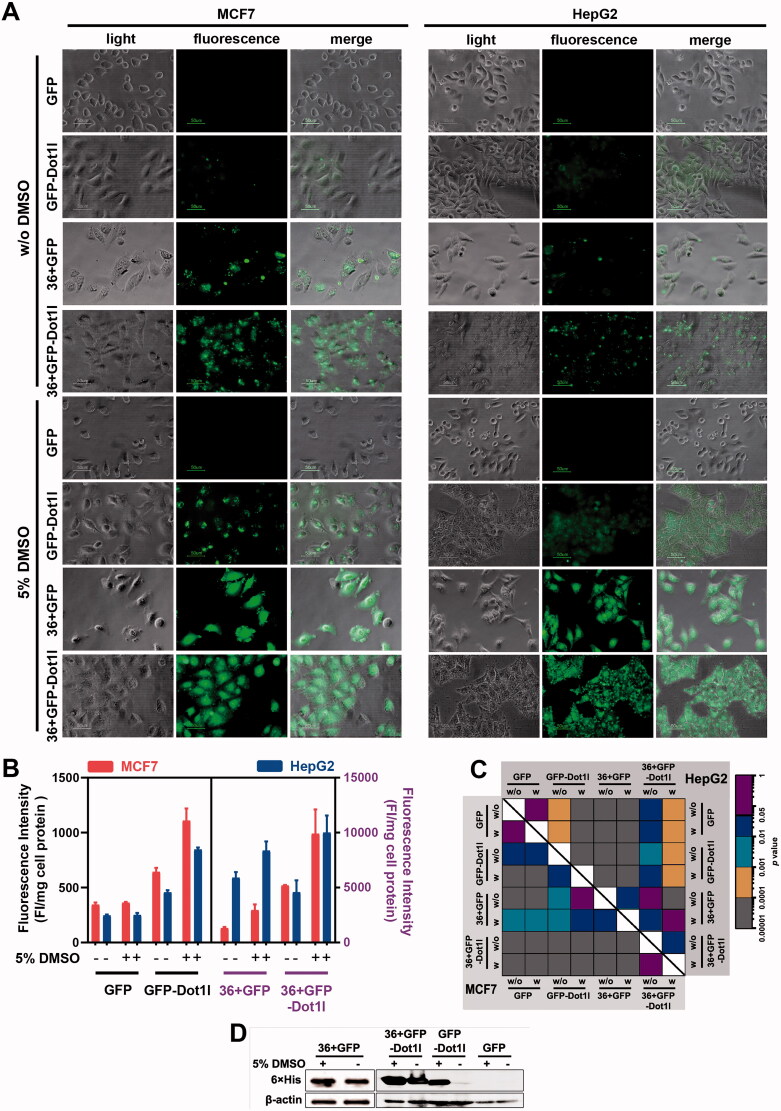
Permeability analysis of recombinant protein in cultured cells. (A) Fluorescence microscopy of MCF7 and HepG2 cells incubated with four different recombinant protein (5 µM) presence or absence of 5% DMSO treatment. (B) Fluorescence intensity quantification MCF7 and HepG2 cells incubated with four different recombinant protein (5 µM) presence or absence of 5% DMSO treatment. The fluorescence intensity quantification from GFP and GFP-Dot1l group were related to the left *y*-axis (black font), 36 + GFP and 36 + GFP-Dot1l group were related to the right *y*-axis (purple font). Red bar indicated MCF7 cell line, blue bar indicated HepG2 cell line. Fluorescence in cell lysate was measured with a multi-mode spectrofluorometer, and fluorescence intensity was normalized by protein concentration. (C) The heatmap of corresponding *p* value between data pairs shown in (B). ANOVA was used to compare the differences between the control and experimental values. (D) Western blot analysis of assessing the availability of proteins inside MCF7 cells. Cells were incubated with recombinant protein for 60 min. After multiple times wash with PBS, total protein was extracted and separated with SDS-PAGE and finally detected by blotting with mouse anti-His antibody.

To examine the permeability of 36 + GFP fused with Dot1l, we examined penetration efficiency of all prokaryotic expressed proteins in cultured MCF7, HepG2, and HSC-T6. As expected in fluorescent microscopy analysis, 36 + GFP-Dot1l can enter cultured MCF7 and HepG2 (Figure 2(A,B)), and HSC-T6 cells (Figures S1B and S1C), and some of punctuate signals indicated that it may be entrapped in endosomes. As indicated in the references (Wang et al., 2010), DMSO can significantly enhance the penetration efficiency of peptide and fusion protein. After 5% DMSO treatment, 36 + GFP-Dot1l can highly efficiently penetrate cultured MCF7 and HepG2 cells and well-distributed in cytosol (Figure 2(A)); although, the penetration efficiency of GFP-Dotl1l was also significantly improved compared to the without DMSO group, it is less efficient than 36 + GFP and 36 + GFP-Dot1l. The fluorescence intensity quantification and statistical analysis of this four-protein treatment is indicated in Figure 2(B,C). To further confirm this four-protein is translocated into cells, immunoblotting was performed after extensive wash before sample collection, and the blotting data are shown in Figure 2(D) indicating that GFP-Dot1l, 36 + GFP, and 36 + GFP-Dot1l were transduced into the cytosol in MCF7 cells. These results suggest Dot1l can further improve the penetration efficiency of GFP and 36 + GFP as well *in vitro* cultured cells.

### 3.3. Recombinant protein has permeability *in vivo*

**Figure 3.**
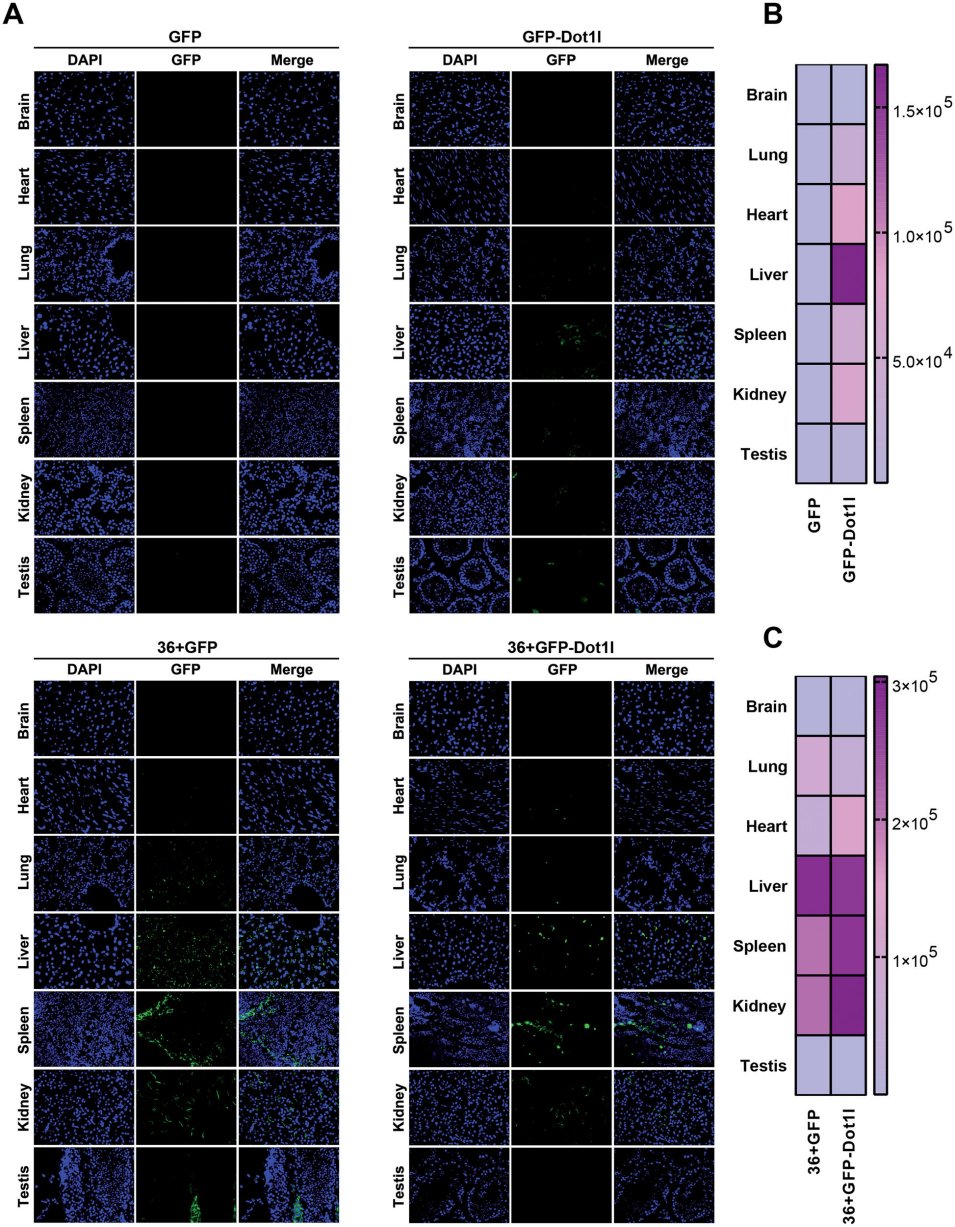
Permeability analysis of recombinant protein *in vivo*. (A) Fluorescence microscopy of GFP in different organs or tissues. For tail vein injection, 200 µg of each recombinant protein were used. After injection for 4 h, mice were sacrificed by deep anesthesia, perfused with 100 ml PBS and then fixed with 4% PFA. Using a cryostat, tissues or organs were cut into 8–10-µm thick slices. Slices mounted on the slide were stained with DAPI. (B) The heat map presenting quantitative analysis of GFP fluorescence intensity in GFP and GFP-Dot1l group. (C) The heat map presenting quantitative analysis of GFP fluorescence intensity in 36 + GFP and 36 + GFP-Dot1l group.

To address whether Dot1l can improve the penetration efficiency of GFP and 36 + GFP *in vivo*, CD1 mice were injected with same amount of purified four proteins via tail vein. After five hours injection, mice were sacrificed and perfused with PBS, and relative tissue and organs were collected. Cryosections of major organs including the brain, heart, lung, liver, spleen, kidney, and testis were made by a cryostat and mounted on slides for further imaging. As shown in Figure 3(A), in comparison with no-signaling GFP group, the slightly fluorescence signaling in GFP-Dot1l group can be observed in lung, heart, spleen, and kidney, although it is higher in liver (up panel of Figure 3(A)), which is consistent with quantification in Figure 3(B). However, in 36 + GFP and 36 + GFP-Dot1l group, a much higher fluorescence signaling can easily be observed, especially in liver, kidney, and spleen (bottom panel of Figure 3(A)). Quantification of fluorescence signaling in 36 + GFP-Dot1l group is higher than that in 36 + GFP group (Figure 3(C)). These results suggested that 36 + GFP and 36 + GFP-Dot1l can efficiently penetrate major organs, and fusion with Dot1l can further improve the efficiency of 36 + GFP protein.

### 3.4. Recombinant protein noncovalently interact with plasmid DNA and form stable protein/plasmid DNA complex

**Figure 4.**
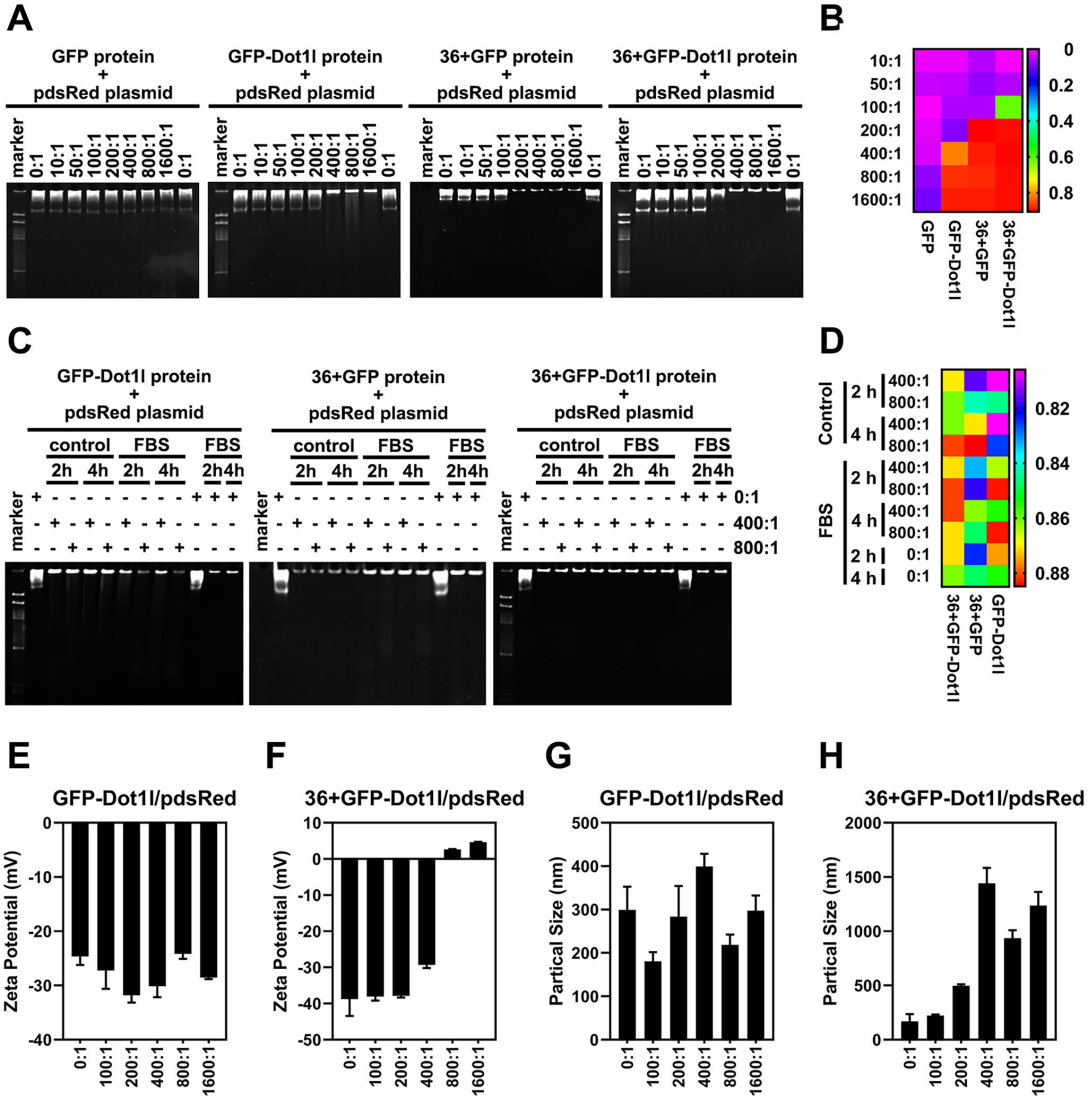
Noncovalent interactions between recombinant protein and plasmid DNA *in vitro*. (A) Native-PAGE electrophoresis (4 h) of the recombinant protein incubated with pdsRed expression plasmid at mol ratios of 10:1 and 1600:1. (B) The heat map presenting quantitative analysis by Image J of gel imaging. DNA band shift migrating in the gel was defined as mobility of recombinant protein/DNA complexes. (C) Effects of serum on the stability of recombinant protein (GFP-Dot1l, 36 + GFP, and 36 + GFP-Dot1l)/plasmid DNA complex with mol ratio of 400:1 and 800:1 for 2 h and 4 h. (D) The heat map presenting quantitative analysis by Image J of gel imaging. DNA band shift migrating in the gel was defined as mobility of recombinant protein/DNA complexes in the presence or absence of serum. (E) Zeta potential of GFP-Dot1l protein/plasmid DNA from mol ratio of 100:1 to 1600:1. (F) Zeta potential of 36 + GFP-Dot1l protein/plasmid DNA from mol ratio of 100:1 to 1600:1. (G) Particle size distribution of GFP-Dot1l protein/plasmid DNA from mol ratio of 100:1 to 1600:1. (H) Particle size distribution of 36 + GFP-Dot1l protein/plasmid DNA from mol ratio of 100:1 to 1600:1.

Before assessing cargo delivery of 36 + GFP and 36 + GFP-Dot1l, we examined the non-covalent interaction between recombinant protein and plasmid DNA by electrophoresis in native-PAGE. As shown in Figure 4(A), different mole rate of protein/pDNA has different mobility. Almost no mobility in GFP/pDNA group was observed, however, at around 200:1 mole rate, mobility of GFP-Dot1l, 36 + GFP, and 36 + GFP-Dot1l were observed. Results from quantification of protein/pDNA mobile distance in Figure 4(B) show that 36 + GFP have stronger interactions than 36 + GFP and GFP-Dot1l group. We also examined serum stability of the recombinant protein/pDNA after 2 h and 4 h incubation (Figure 4(C,D)) and observed very slightly degradation of pDNA in GFP-Dot1l and 36 + GFP group, but it was not present in 36 + GFP-Dot1l. Furthermore, zeta potential (Figure 4(E,F)) and particle size (Figure 4(G,H)) of recombinant protein/pDNA were also determined. GFP-Dot1l/pDNA have no significant difference in net negative charge in all mole rate group (Figure 4(E)), while 36 + GFP-Dot1l/pDNA have net positive charge start from mole rate of 800:1 (Figure 4(F)). The particle size of GFP-Dot1l/pDNA is below 500 nm (Figure 4(G)), however, the size of 36 + GFP-Dot1l/pDNA complex is above 500 nm start from mole rate of 200:1 (Figure 4(H)). These results suggest that the recombinant protein can noncovalently interact with plasmid DNA and form stable protein/plasmid DNA complex.

### 3.5. No observable cytotoxicity of recombinant protein/plasmid DNA complex

**Figure 5.**
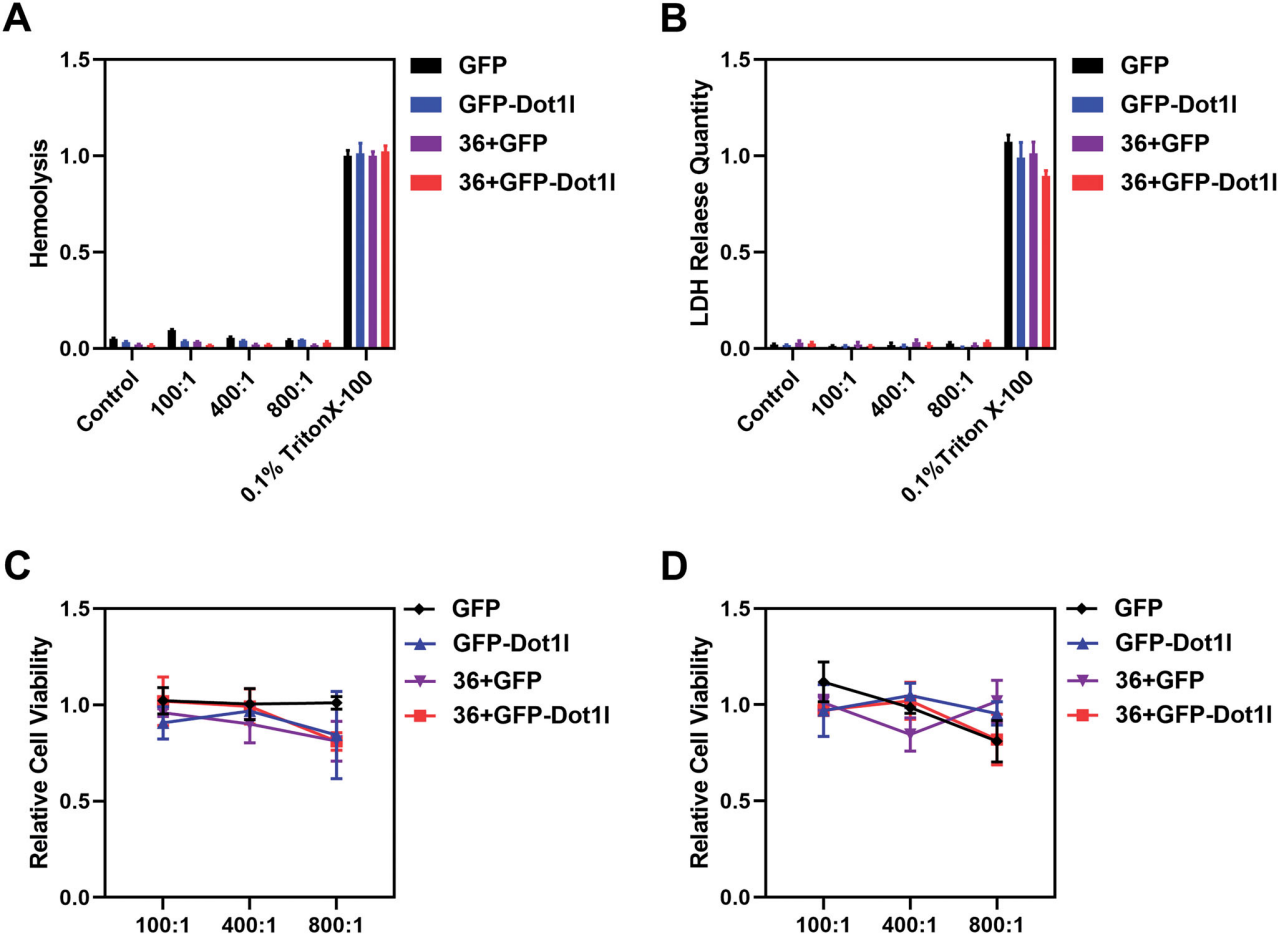
Cytotoxicity of recombinant protein/plasmid DNA complexes. (A) Hemolysis of GFP, GFP-Dot1l, 36 + GFP, and 36 + GFP-Dot1l recombinant proteins/plasmid from the ratio of 100:1 to 800:1. (B) LDH release quantitative analysis of GFP, GFP-Dot1l, 36 + GFP, and 36 + GFP-Dot1l recombinant proteins/plasmid from the ratio of 100:1 to 800:1. (C) MTT analysis of GFP, GFP-Dot1l, 36 + GFP, and 36 + GFP-Dot1l recombinant proteins/plasmid from the ratio of 100:1 to 800:1 after 24 h in cultured MCF7 cells. (D) MTT analysis of GFP, GFP-Dot1l, 36 + GFP, and 36 + GFP-Dot1l recombinant proteins/plasmid from the ratio of 100:1 to 800:1 after 48 h in cultured MCF7 cells.

After the recombinant protein/pDNA complex stability evaluation, the assessment of cytotoxicity and safety of protein/pDNA complex at different mole rate were conducted. Hemolysis assay (Figure 5(A)) and LDH release assay (Figure 5(B)) were used to examine the safety of protein/pDNA complex. We did not observe RBC membrane damage (Figure 5(A)) and LDH release from cultured MCF7 cells (Figure 5(B)). Moreover, we also performed MTT assay to examine the cytotoxicity of recombinant protein/pDNA complex at 24 h (Figure 5(C)) and 48 h (Figure 5(D)) in cultured MCF7 cells, but no significant MCF7 cell growth inhibition was observed. These results indicated that the recombinant protein/pDNA complex is safe.

### 3.6. Recombinant protein mediate plasmid delivery efficiently in cultured cells

**Figure 6.**
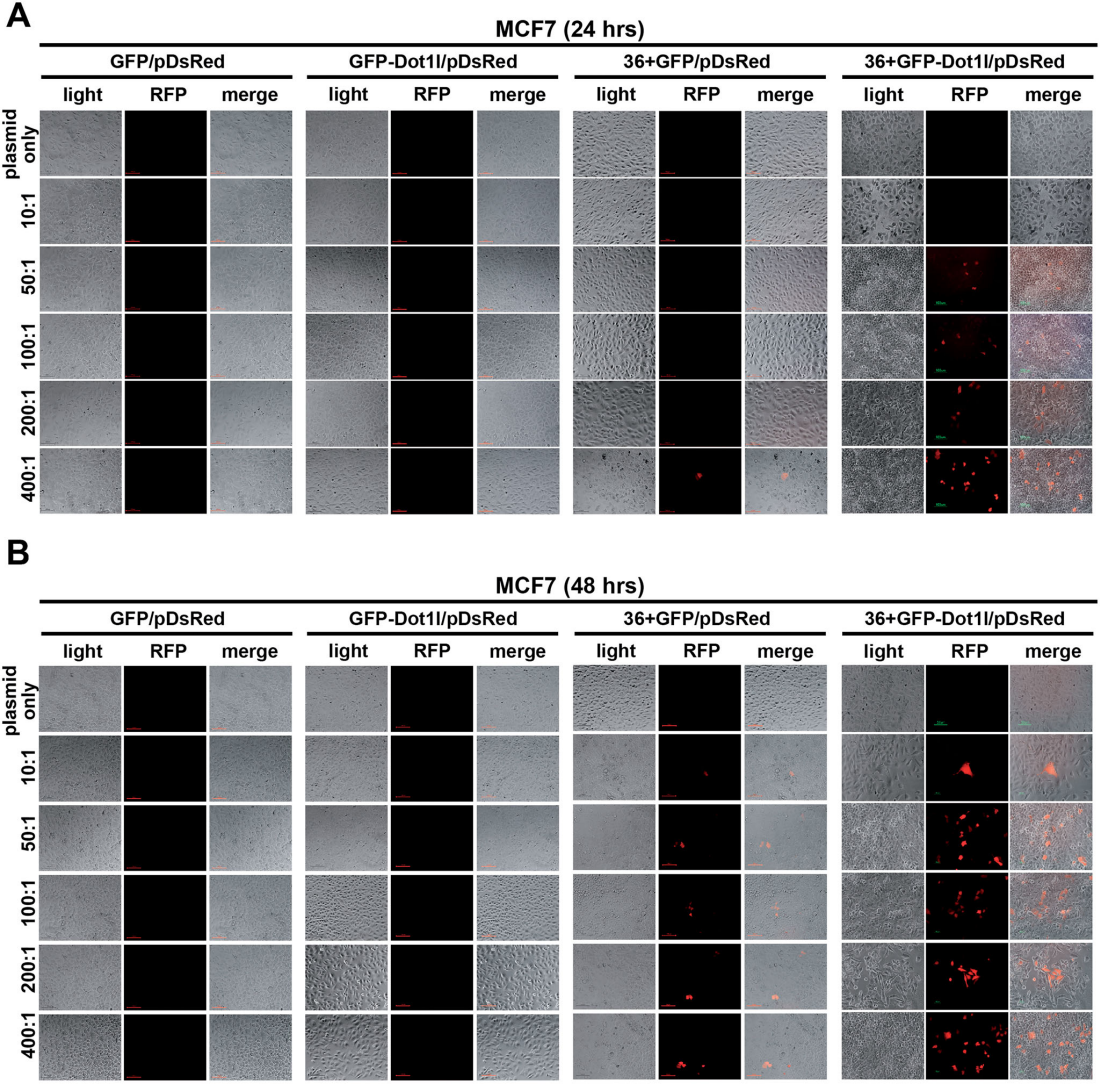
Recombinant protein mediated DsRed expression plasmid DNA delivery *in vitro*. (A) Fluorescence images of recombinant proteins mediated pdsRed expression plasmid DNA delivery into cultured MCF7 cells from mol ratio of 10:1 to 400:1 after 24 h. (B) Fluorescence images of recombinant proteins mediated pdsRed expression plasmid DNA delivery into cultured MCF7 cells from mol ratio of 10:1 to 400:1 after 48 h.

Next, we evaluated the plasmid transfection mediated by recombinant protein *in vitro* cultured MCF7 cells (Figure 6, Figures S2, S3, and S6) and HSC-T6 cells (Figures S4, S5, and S7). Before we examined the DsRed-expressing plasmid expression, fusion proteins’ transduction in the presence of 5% DMSO treatment was conducted, as shown in Figure S2, and green fluorescence is still can be observed in GFP-Dot1l, 36 + GFP, and 36 + GFP-Dot1l after 24 (Figure S2A) or 48 (Figure S2B) hours treatment. However, green fluorescence in GFP-Dot1l/pDNA group is much weaker 36 + GFP/pDNA and 36 + GFP-Dot1l/pDNA group. Then, we examined the red fluorescence from DsRed-expressing plasmid expression. As shown in Figure 6, we did not observe any DsRed-expressing red fluorescence signaling in GFP/pDNA and GFP-Dot1l/pDNA group with different mole rate after 24 h (Figure 6(A)) or 48 h (Figure 6(B)) transfection. Although, we observed very few cells have red fluorescence signaling in 36 + GFP/pDNA group, the 36 + GFP-Dot1l/pDNA group with different mole rate have an apparent higher transfection. Transfection mediated by higher mole rate of recombinant protein was also evaluated, as shown in Figure S3. However, we did not observe further improvement. We also examined the efficiency of transfection meditated by recombinant protein in the presence of 5% DMSO treatment in cultured HSC-T6 cells (Figures S4 and S5). However, even 36 + GFP-Dot1l/pDNA group is apparently less efficient compared with MCF7 cells. As noted, without 5% DMSO treatment, although 36 + GFP and 36 + GFP-Dot1l can mediate DsRed-expressing plasmid transfection in MCF7 cells (Figure S6) and HSC-T6 cells (Figure S7), their transfection efficiency is much lower than that without DMSO treatment. These results suggested that 36 + GFP and 36 + GFP-Dot1l can mediate plasmid transfection *in vitro* cultured cells, but their transfection efficiency is limited, and different cell lines may result in variations.

### 3.7. Recombinant protein can mediate plasmid delivery *in vivo* in mouse model

**Figure 7.**
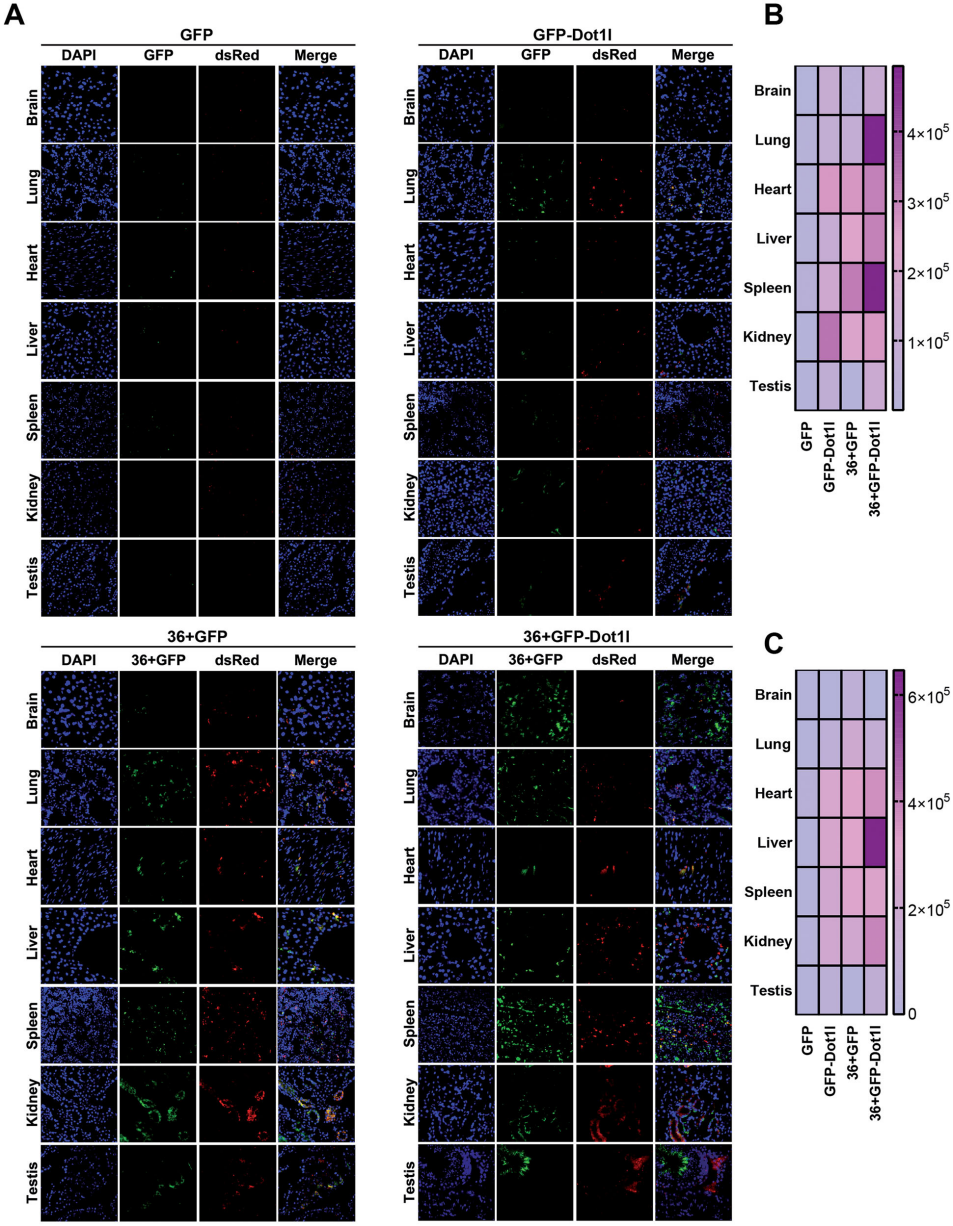
Recombinant protein mediated DsRed expression plasmid DNA delivery *in vivo*. (A) Fluorescence microscopy of GFP and RFP in different organs or tissues. For tail vein injection, 100 µg of each recombinant protein–plasmid complex at mole ratio of 800:1 was used. After injection for 48 h, mice were sacrificed by deep anesthesia, perfused with 100 ml PBS and then fixed with 4% paraformaldehyde (PFA). Using a cryostat, tissues or organs were cut into 8–10 µm thick slices. Slices mounted on the slide were stained with DAPI and observed under fluorescence microscope. (B) The heat map presenting quantitative analysis of GFP fluorescence intensity in four recombinant protein group. (C) The heat map presenting quantitative analysis of RFP fluorescence intensity in four recombinant protein group.

As shown above, 36 + GFP and 36 + GFP-Dot1l can mediate but with limitation in plasmid transfection *in vitro*. To address whether recombinant protein can meditate plasmid delivery *in vivo*, we prepared recombinant protein/pDNA complex, and injected them into CD1 mice via tail vein. The samples process procedure is the same with recombinant protein transduction assay shown above. As expected, we cannot observe green and red fluorescence in GFP/pDNA group (Figure 7(A)), although very few green (Figure 7(B)) and red signaling (Figure 7(C)) can be observed in GFP/pDNA group. 36 + GFP and 36 + GFP-Dot1l group have much higher transfection efficiency. These indicated that 36 + GFP and 36 + GFP-Dot1l can efficiently penetrate different organs (Figure 7(A,B)), and further mediate DsRed plasmid transfection and expression (Figure 7(A,C)). More interestingly, 36 + GFP-Dot1l can across the blood brain barrier and mediate higher transfection efficiency in different tissues or organs. Additionally, 36 + GFP-Dot1l/pDNA group has higher transfection than 36 + GFP/pDNA group (Figure 7(C)). These results suggest that recombinant protein can mediate DsRed-expressing plasmid delivery *in vivo* in mouse model.

## 4. Discussion

Previous studies suggested that supercharging with cationic Lys/Arg/His residues allow supercharging proteins to control their properties such as temperature resistance, unusual resistance to aggregation and catalytic activity (Prasse et al., 2014; Ma et al., 2020). Although published reports indicated that 36 + GFP can enter the cultured cells, and mediate fusion protein and siRNA fragment (McNaughton et al., 2009) *in vitro* and *in vivo* (Li et al., 2015; Zhang et al., 2020), we noted that the efficiency of 36 + GFP based delivery was not high, which may be entrapped in the endosome. Moreover, even though cationic peptide fused with 36 + GFP can facilitate the endosomal escape, it is still limited (Li et al., 2015). To identify a way to increase the efficiency of 36 + GFP based delivery, according to our previous findings (Geng et al., 2020), we designed 36 + GFP-Dot1l fusion protein. We examined the penetration efficacy of fusion protein *in vitro* and *in vivo*, after packaged with plasmid DNA, and further investigated its ability of mediating plasmid transfection *in vitro* and *in vivo*. We found that the efficacy of *in vitro* and *in vivo* transfection mediated by 36 + GFP-Dot1l fusion protein is significantly improved than 36 + GFP itself. These findings suggested that CPP-Dot1l can synergically promote 36 + GFP mediate plasmid delivery *in vitro* and *in vivo*.

Our studies also suggested although 36 + GFP can significantly enter cells and tissues compared with GFP protein, the penetration efficiency of 36 + GFP is still limited. Following our previous studies (Ma et al., 2015; Wang et al., 2016a, 2016b, 2016c; Zhou et al., 2017; Ding et al., 2019; Zhang et al., 2019; Chen et al., 2021; Guo et al., 2021), we conducted 5% DMSO treatment in cultured cells, and found that the efficiency of 36 + GFP mediated transduction itself and its mediated transfection were significantly increase thus these results are consistent with our previous published studies. These data indicated that penetration enhancer application is another alternative approach to enhance the penetration and relative delivery efficiency of peptide or supercharged proteins- based delivery vectors.

Additionally, supercharged 36 + GFP protein itself can penetrate *in vitro* cultured cells and different organ or tissue *in vivo*. However, its penetration efficiency is low while after fused with Dot1l, its penetration efficiency is significantly improved. Although previous study suggested GFP can be mutated with 48 and 72 net positive charged residues, little is known about their application. Our previous study reported that Dot1l can significantly enhance the penetration efficiency of GFP protein (Geng et al., 2020). Therefore, we further used positive charge residue enriched Dot1l fragment to fuse with 36 + GFP to further improve its penetration efficiency. Our *in vivo* analysis of 36 + GFP mediated transduction and transfection indicated that CPPs like Dot1l can further be used to enhance the penetration and relative delivery efficiency of supercharged proteins-based delivery vectors.

Although our study indicated that 36 + GFP itself is inefficient to cross the blood tissue barrier such as the blood brain barrier and blood testes barrier, and as well in 36 + GFP-Dot1l, 36 + GFP-Dot1l/pDNA can have a relative higher efficiency in the brain than 36 + GFP. This may be due to the increase of zeta potential which is a key parameter to facilitate the protein-based delivery (Mangla et al., 2020). However, DsRed expression in the brain is still very low which may be due to the promoter of the plasmid and different tissue (Regan et al., 2007). It is worth noting that 36 + GFP-Dot1l based transfection is significantly improved compared with 36 + GFP.

In summary, supercharged 36 + GFP based protein delivery can be further improved by fusion with peptide CPP-Dot1l or together with penetration enhancer, which may support supercharged 36 + GFP protein based macromolecular delivery *in vitro* and *in vivo* in future.

## Supplementary Material

Supplemental Material

## Data Availability

All data generated or analyzed during current study are included in this article and its supplementary materials.
