# Supplementary material for: Improved transfer efficiency of supercharged 36 + GFP protein mediate nucleic acid delivery
Source: Drug Deliv. 2022 Jan 25;29(1):386–98. doi: 10.1080/10717544.2022.2030430 (PMC8794074; doi:10.1080/10717544.2022.2030430)
Supplement: Supplemental Material [file IDRD_A_2030430_SM7019.docx]

**Table S1 CPP-Dot1l DNA and protein sequence**

| 10 20 30 40 50 60 |
| --- |
| 1 ATGGGCAGCAGCCATCATCATCATCATCACAGCAGCGGCCTGGTGCCGCGCGGCAGCCAT |
| 1 M G S S H H H H H H S S G L V P R G S H |
|  |
| 70 80 90 100 110 120 |
| 61 ATGAAAGGTGAACGTCTGTTTCGTGGTAAAGTACCGATCTTAGTGGAATTAAAGGGCGAC |
| 21 M K G E R L F R G K V P I L V E L K G D |
|  |
| 130 140 150 160 170 180 |
| 121 GTGAACGGTCATAAATTTAGCGTGCGCGGCAAAGGCAAAGGTGACGCTACCCGTGGTAAA |
| 41 V N G H K F S V R G K G K G D A T R G K |
|  |
| 190 200 210 220 230 240 |
| 181 TTGACCCTGAAGTTTATTTGCACAACAGGCAAATTACCCGTTCCGTGGCCCACCTTAGTG |
| 61 L T L K F I C T T G K L P V P W P T L V |
|  |
| 250 260 270 280 290 300 |
| 241 ACCACCCTGACCTATGGCGTTCAGTGCTTCAGTCGTTACCCTAAACACATGAAACGTCAC |
| 81 T T L T Y G V Q C F S R Y P K H M K R H |
|  |
| 310 320 330 340 350 360 |
| 301 GATTTTTTCAAATCAGCCATGCCTAAAGGATATGTTCAAGAGCGTACAATCAGCTTCAAG |
| 101 D F F K S A M P K G Y V Q E R T I S F K |
|  |
| 370 380 390 400 410 420 |
| 361 AAGGATGGCAAATATAAAACGCGTGCGGAAGTGAAATTTGAAGGCCGCACATTAGTAAAT |
| 121 K D G K Y K T R A E V K F E G R T L V N |
|  |
| 430 440 450 460 470 480 |
| 421 CGTATCAAACTGAAAGGTCGTGACTTCAAAGAAAAAGGCAACATTTTAGGCCATAAACTG |
| 141 R I K L K G R D F K E K G N I L G H K L |
|  |
| 490 500 510 520 530 540 |
| 481 CGTTATAACTTTAATTCTCATAAGGTGTATATTACGGCCGATAAACGCAAGAATGGTATC |
| 161 R Y N F N S H K V Y I T A D K R K N G I |
|  |
| 550 560 570 580 590 600 |
| 541 AAGGCAAAATTCAAAATTCGCCATAACGTGAAAGACGGCAGCGTTCAATTAGCGGATCAT |
| 181 K A K F K I R H N V K D G S V Q L A D H |
|  |
| 610 620 630 640 650 660 |
| 601 TATCAACAAAACACGCCGATTGGTCGCGGGCCTGTACTGTTACCTCGCAACCACTACCTG |
| 201 Y Q Q N T P I G R G P V L L P R N H Y L |
|  |
| 670 680 690 700 710 720 |
| 661 AGCACCCGTTCTAAACTGAGCAAAGATCCGAAAGAAAAACGCGATCACATGGTTCTGTTA |
| 221 S T R S K L S K D P K E K R D H M V L L |
|  |
| 730 740 750 760 770 780 |
| 721 GAATTCGTGACCGCTGCAGGCATTAAGCACGGACGCGACGAACGCTACAAGCTCGAGAAA |
| 241 E F V T A A G I K H G R D E R Y K L E K |
|  |
| 790 800 810 820 830 840 |
| 781 GCCCGCAAGAAGAAGCTAAACAAGAAGGGGAGGAAGATGGCTGGCCGCAAGCGCGGGCGC |
| 261 A R K K K L N K K G R K M A G R K R G R |
|  |
| 850 |
| 841 CCCAAGAAGTAA |
| 281 P K K * |
